# The human phrenic nerve serves as a morphological conduit for autonomic nerves and innervates the caval body of the diaphragm

**DOI:** 10.1038/s41598-018-30145-x

**Published:** 2018-08-03

**Authors:** Thomas J. M. Verlinden, Paul van Dijk, Andreas Herrler, Corrie de Gier - de Vries, Wouter H. Lamers, S. Eleonore Köhler

**Affiliations:** 1 Department of Anatomy & Embryology, Faculty of Health, Medicine and Life Sciences, Maastricht University, Maastricht, The Netherlands; 2 Department of Medical Biology, Academic Medical Center, University of Amsterdam, Amsterdam, The Netherlands; 3 Tytgat Institute for Liver and Intestinal Research, Academic Medical Center, University of Amsterdam, Amsterdam, The Netherlands

## Abstract

Communicating fibres between the phrenic nerve and sympathetic nervous system may exist, but have not been characterized histologically and immunohistochemically, even though increased sympathetic activity due to phrenic nerve stimulation for central sleep apnoea may entail morbidity and mortality. We, therefore, conducted a histological study of the phrenic nerve to establish the presence of catecholaminergic fibres throughout their course. The entire phrenic nerves of 35 formalin-fixed human cadavers were analysed morphometrically and immunohistochemically. Furthermore, the right abdominal phrenic nerve was serially sectioned and reconstructed. The phrenic nerve contained 3 ± 2 fascicles in the neck that merged to form a single fascicle in the thorax and split again into 3 ± 3 fascicles above the diaphragm. All phrenic nerves contained catecholaminergic fibres, which were distributed homogenously or present as distinct areas within a fascicle or as separate fascicles. The phrenicoabdominal branch of the right phrenic nerve is a branch of the celiac plexus and, therefore, better termed the “phrenic branch of the celiac plexus”. The wall of the inferior caval vein in the diaphragm contained longitudinal strands of myocardium and atrial natriuretic peptide-positive paraganglia (“caval bodies”) that where innervated by the right phrenic nerve.

## Introduction

The phrenic nerve arises from the anterior roots of the third to fifth cervical nerves and is known to innervate the diaphragm^1,2^. Accessory fibres from cervical segments that join the phrenic nerve are common and mainly originate from the subclavian nerve, the ansa cervicalis, and the sternohyoid nerve^3^. Communicating fibres between the sympathetic trunk and the phrenic nerve in the cervical region were already described by Luschka in 1853^4^. Some phrenic fibres may contribute to the cardiac plexus^5^, but the evidence is limited^6^. Communications between the phrenic nerve and the ansa subclavia, a structure known to contribute to the inferior cervical sympathetic cardiac nerve^7^, have been described more often^5,6,8^. Additionally, a continuation of the right phrenic nerve towards the aortic autonomic plexus in the abdomen, the so-called phrenicoabdominal branch, is often reported^7,9,10^. This branch contains one or several ganglia that stain positive for tyrosine hydroxylase (TH)^11^, indicating catecholaminergic signal transmission.

Recently, the phrenic nerve has become the target of interventions that aim to regulate breathing patterns by electrical nerve stimulation^12^. An example is the treatment of central sleep apnoea^13,14^. For such an application of phrenic nerve stimulation, it is important to be aware of all components of the phrenic nerve, as they are also stimulated in such procedures. The presence of a catecholaminergic component within the phrenic nerve warrants particular attention when phrenic nerve stimulation is applied, because increased sympathetic activity is associated with increased mortality in patients with central sleep apnoea^15–17^.

The earlier descriptions of communicating branches between the phrenic nerve and the sympathetic nervous system were based on macroscopic dissections, which do not permit distinction between nerves and connective tissue strands. Furthermore, the dissectional approach does not reveal whether sympathetic (catecholaminergic) fibres are present in such communications. We, therefore, conducted a detailed histological study to establish the presence of catecholaminergic fibres in the phrenic nerve throughout its course from its cervical roots to its connection with the celiac plexus. Since phrenic nerve stimulation can be performed on both sides and at different levels of the nerve, we also looked at morphological differences along the course of the phrenic nerve and compared left and right phrenic nerves at different levels.

## Methods

Nerve tissue was harvested from thirty-five (16 female, 19 male) formalin-fixed cadavers between 58 and 101 ($\bar{{\rm{x}}}\,$ = 84 ± 11) years of age from the body donation program of the Department of Anatomy and Embryology, Maastricht University. The tissue donors gave their informed and written consent to the donation of their body for teaching and research purposes as regulated by the Dutch law for the use of human remains for scientific research and education (Wet op de Lijkbezorging, 1991). Accordingly, a handwritten and signed codicil from the donor posed when still alive and well, is kept at the Department of Anatomy and Embryology Faculty of Health, Medicine and Life Sciences, Maastricht University, Maastricht, The Netherlands. The bodies were preserved by intra-arterial infusion with 10 L fixative (composition (v/v): 96% ethanol (21%), glycerin (21%), 36% formaldehyde (2%), water (56%), and 2.4 g/L thymol), followed by 4 weeks of fixation in 96% ethanol (20%), 36% formaldehyde (2%) and water (78%). Samples were only taken from bodies without signs of previous surgical interventions on neck, thorax or abdomen.

### Phrenic nerve sampling

**Figure 1:**
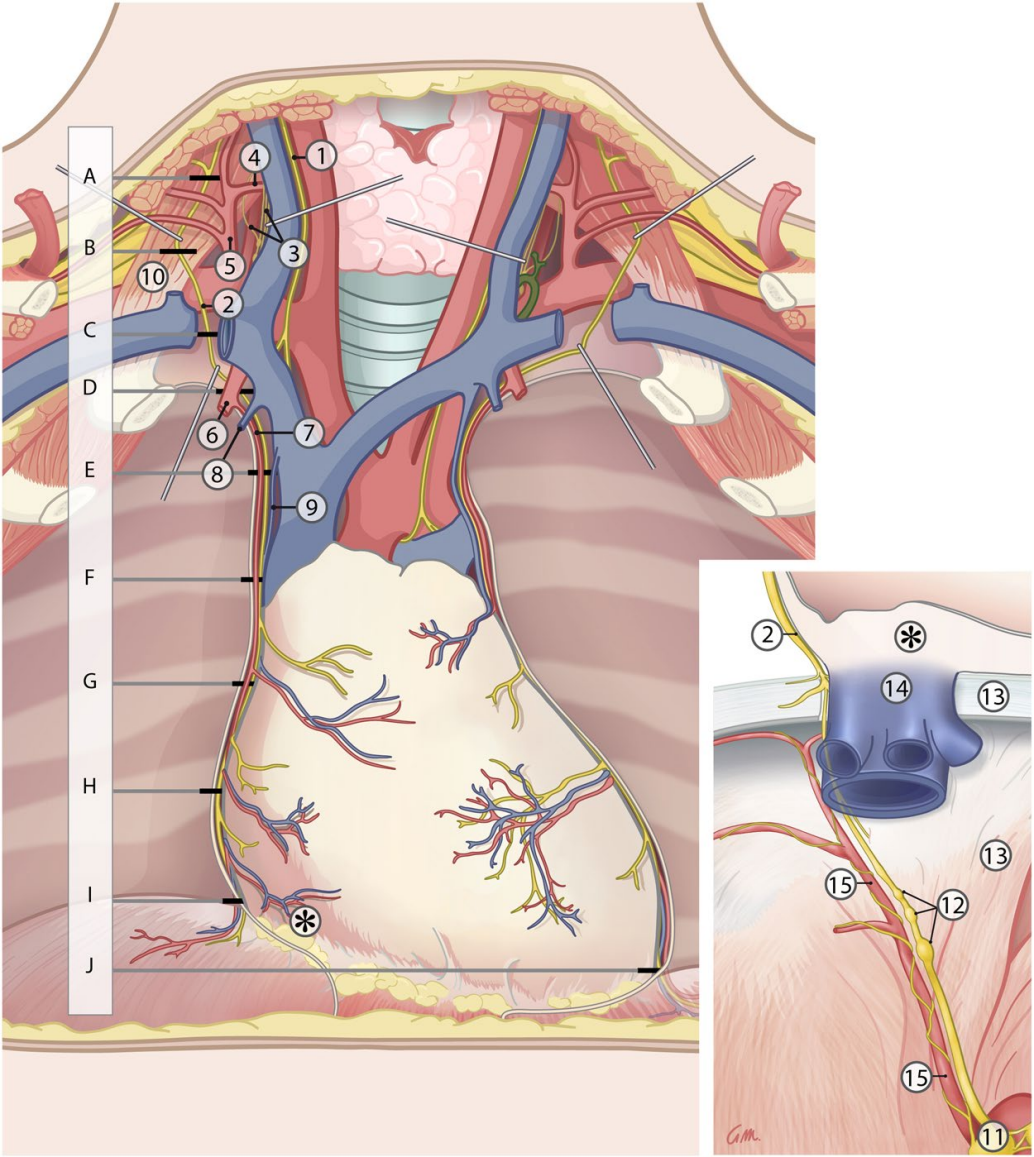
Sampling of the phrenic nerve. (**A**–**I**) Indicate the sampling sites of the respective cervical and thoracic samples. (**J**) Indicates the position of the extra thoracic sampling site for the left phrenic nerve. The inset shows a magnification of the peridiaphragmatic portion of the phrenic nerve (the asterisks indicate corresponding sites on the pericard). 1: Vagus nerve; 2: phrenic nerve; 3: cervical sympathetic plexus; 4: inferior thyroid artery; 5: thyrocervical trunk; 6: internal thoracic artery (cut); 7: pericardiophrenic artery; 8: internal thoracic vein; 9: pericardiophrenic vein; 10: anterior scalene muscle; 11: celiac ganglion and plexus; 12: phrenic ganglia; 13: diaphragm; 14: inferior caval vein; 15: inferior phrenic artery. Note the connection between the right phrenic nerve and the celiac autonomic nerve plexus. Illustration drawn by Greet Mommen.

The cervical and thoracic portions of the phrenic nerve with surrounding connective tissue and accompanying pericardiophrenic vessels were collected and subdivided into levels A to I (−J on the left side), as depicted in Fig. 1. To investigate whether an abdominal branch of the phrenic nerve extends to the celiac plexus (inset Fig. 1), the abdominal phrenic nerves, including the part that traversed the diaphragm, were collected and embedded ‘en bloc’ for further histological processing. Furthermore, periarterial tissue accompanying left and right inferior phrenic arteries was collected to establish whether or not the phrenic nerve contributes to the nerve plexus accompanying the inferior phrenic arteries.

### Histological processing

All cervical and thoracic nerve samples were cut transversally into two parts. The upper parts were post-fixed overnight in 1% osmium tetroxide (OsO_4_)/phosphate-buffered saline (PBS) and then embedded in paraffin. The lower parts, the abdominal samples, and the ‘en bloc’ samples of diaphragm and abdominal phrenic nerve were subjected to standard paraffin embedding. Five micrometre-thick sections were prepared of all samples with a Leica 2245 microtome. Mounted sections were used for haematoxylin and eosin (HE) staining or immunohistochemistry. To determine the intradiaphragmatic course of the phrenic nerves, the ‘en bloc’ samples were sectioned every 250 µm (4 sections per mm). For reconstruction, one abdominal phrenic nerve was sectioned completely into 5 µm consecutive transverse sections resulting in 7,500 slides.

To determine the surface area of catecholaminergic nerve fibres, antibodies against tyrosine hydroxylase (TH; 1:1,000, Abcam AB112, Cambridge, UK) and dopamine ß-hydroxylase (DBH; 1:150, Abcam AB109112) were used. Antibodies against protein gene product 9.5 (PGP9.5; 1:100, Biotrend APG0714, Cologne, Germany) (a ubiquitin C-terminal hydroxylase, highly specific for neurons), S100 protein (S100; 1:1,000, Dako Z0311, Glostrup, Denmark) (used for identifying axons and dendrites), choline acetyltransferase (ChAT; 1:50, Merck, AB144P, Darmstadt, Germany) and vesicular acetylcholine transporter (VAChT) (1:2,000, MBL-Sanbio BMP 048, Japan) were used to determine the surface area of (cholinergic) nerve tissue. The antibody against natriuretic peptide A (NPPA) was purchased from Campro Scientific (RGG 9103, Veenendaal, The Netherlands; 1:200). For the detection of cardiac muscle tissue, a SERCA2a antiserum that was raised in rabbits against the BSA-coupled C terminal SERCA2a peptide NYLEPAILE (1:1,000) was used^18^.

Complete images of selected large slides were digitized with an Olympus BX61 scanning microscope and the DOTSLIDE program (Olympus, Zoeterwoude, The Netherlands). AMIRA software (version 6.0; base package; FEI Visualization Sciences Group Europe, Mérignac Cédex, France) was used to generate 3D reconstructions after image loading, alignment and segmentation^19^.

### Morphometric analysis

Slides were photographed with a Leica (type DMRD) photomicroscope^20^. Surface areas of myelinated axons, specifically stained axons, and the entire surface of the nerve within the perineurium, including its supporting tissue, were measured with Leica Qwin v.3.5.1 analysis software at 10x magnification. Furthermore, the number of nerve fascicles was counted. Two persons independently determined whether staining exceeded background levels. The average of these values was used as threshold.

Statistical analysis was performed with Graphpad Prism v6.0 software. Data were tested for normality with the Shapiro-Wilk normality test. Comparisons were made by Student’s t-tests and one-way ANOVA followed by Bonferroni post-hoc tests. Data are presented as means ± SD. Individually presented data are displayed after Savitzky-Golay filtering. P-values < 0.05 were considered as statistically significant.

## Results

### Cervical and thoracic findings

#### Surface area measurements

Nineteen cadavers were analysed. The mean surface area of the right and left nerves excluding epi- and perineurium was 0.35 ± 0.02 and 0.29 ± 0.01 mm^2^_,_ yielding diameters of 1.2 and 1.1 mm, respectively. No significant differences in surface area along the proximo-distal course of the nerve were observed for either the right or left nerve (P ≥ 0.71). Furthermore, no significant differences in surface area between corresponding levels of the left and right nerves were detected (all P ≥ 0.09).

#### Myelination

Myelinated neurons occupied 26 ± 1% and 29 ± 1% of the total surface areas of the right and left nerves, respectively. No significant differences in myelination were observed along the proximo-distal course of the right and left nerve (P ≥ 0.10), or between corresponding levels of the right and left nerves (all P ≥ 0.35).

#### Fascicles

**Figure 2:**
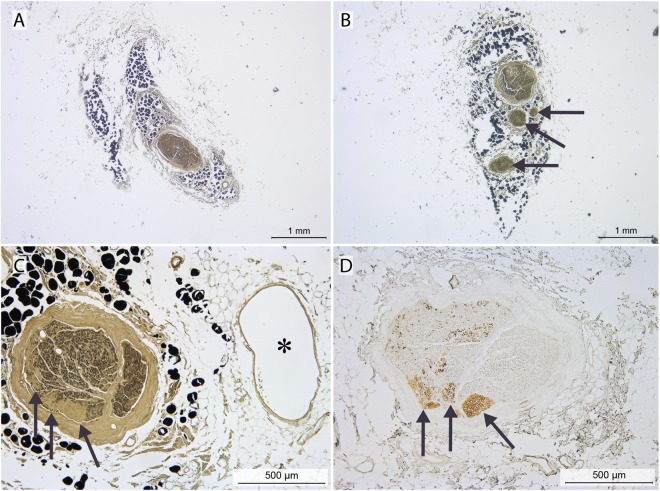
Morphological characteristics of the phrenic nerve in neck and thorax. (**A**–**C**) Osmium-tetroxide staining. Black dots represent osmium-stained adipocytes within the epineurium. (**D**) Tyrosine Hydroxylase (TH) staining. (**A**–**C**) Show the phrenic nerve as: a single fascicle at cervical sampling site C (**A**) several fascicles (arrows) in the cervicothoracic transition area at sampling site D (**B**) or a single fascicle at thoracic sampling site E (**C**). Note the close relationship of the phrenic nerve with the pericardiophrenic vein (indicated by asterisk). (**D**) Adjacent section of C, stained for the presence of TH (arrows). Note that TH-positive areas are OsO_4_-negative (arrows).

**Figure 3:**
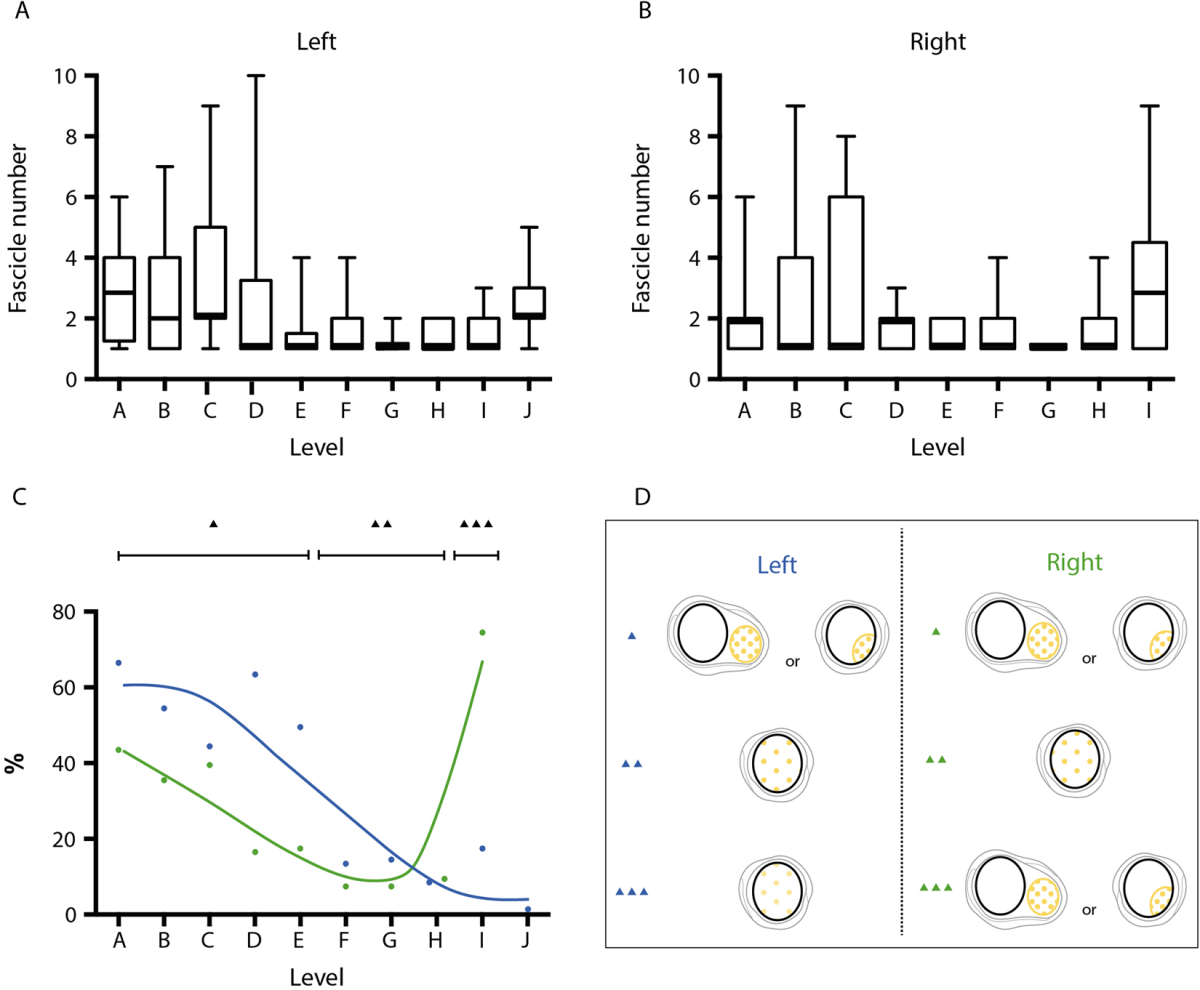
(Tyrosine hydroxylase-positive-) Fibres and fascicles within the phrenic nerve at the different levels. (**A**,**B**) Number of fascicles. Median, first and third quartile, and range are shown as boxplots, A left and B right phrenic nerve. (**C**) Percentage of TH-positive fibres (blue: left, green: right) presenting as distinct areas. (**D**) TH-positive fibres (yellow) presented as distinct fascicles or as distinct areas within the phrenic perineurium or epineurium, respectively (most often seen between levels A and E and on the right side only at level I) as opposed to a homogenous distribution of TH-positive fibres (most often seen between levels F and J except for level I on the right side; for levels confer Fig. 1). Triangles in (**C** and **D**) are corresponding to the levels, with one triangle indicating levels A–E, two triangles levels F–H and 3 triangles levels I-J, respectively. Illustration drawn by Greet Mommen.

In the cervical area, the phrenic nerves contained 3 ± 2 fascicles (as defined by having a distinct perineurium; range: 1–10 fascicles; Figs 2A,B and 3A,B) that merged to form the single fascicle usually seen in the thorax (range: 1–4; Figs 2C and 3A). Just cranial to the diaphragm the number of fascicles increased to 3 ± 3 (range 1–9; Fig. 3A,B). No significant differences were found between left and right phrenic nerves (all P ≥ 0.08).

#### Expression of Tyrosine Hydroxylase

Tyrosine hydroxylase (TH)-positive fibres were found in all phrenic nerves studied (Fig. 2D), occupied 1.6 ± 0.5 and 2.0 ± 0.6% of the total nerve surface area of the right and left nerves, respectively, and were present in the nonmyelinated areas (Fig. 2C). The size of the TH-positive areas was similar along the proximo-distal course of the nerve for both the right (P = 0.63) and left nerves (P ≥ 0.63), and between corresponding levels of the left and right nerves (all P ≥ 0.09).

#### TH-positive fibres and fascicles

TH-positive fibres were either distributed homogeneously or presented as distinct areas or fascicles within the phrenic perineurium or epineurium, respectively (Fig. 3D). The distinct areas were seen most frequently in the left and right cervical and the right thoracic region just above the diaphragm (Fig. 3C).

### Findings near the diaphragm

#### Intradiaphragmatic course of the phrenic nerves

**Figure 4:**
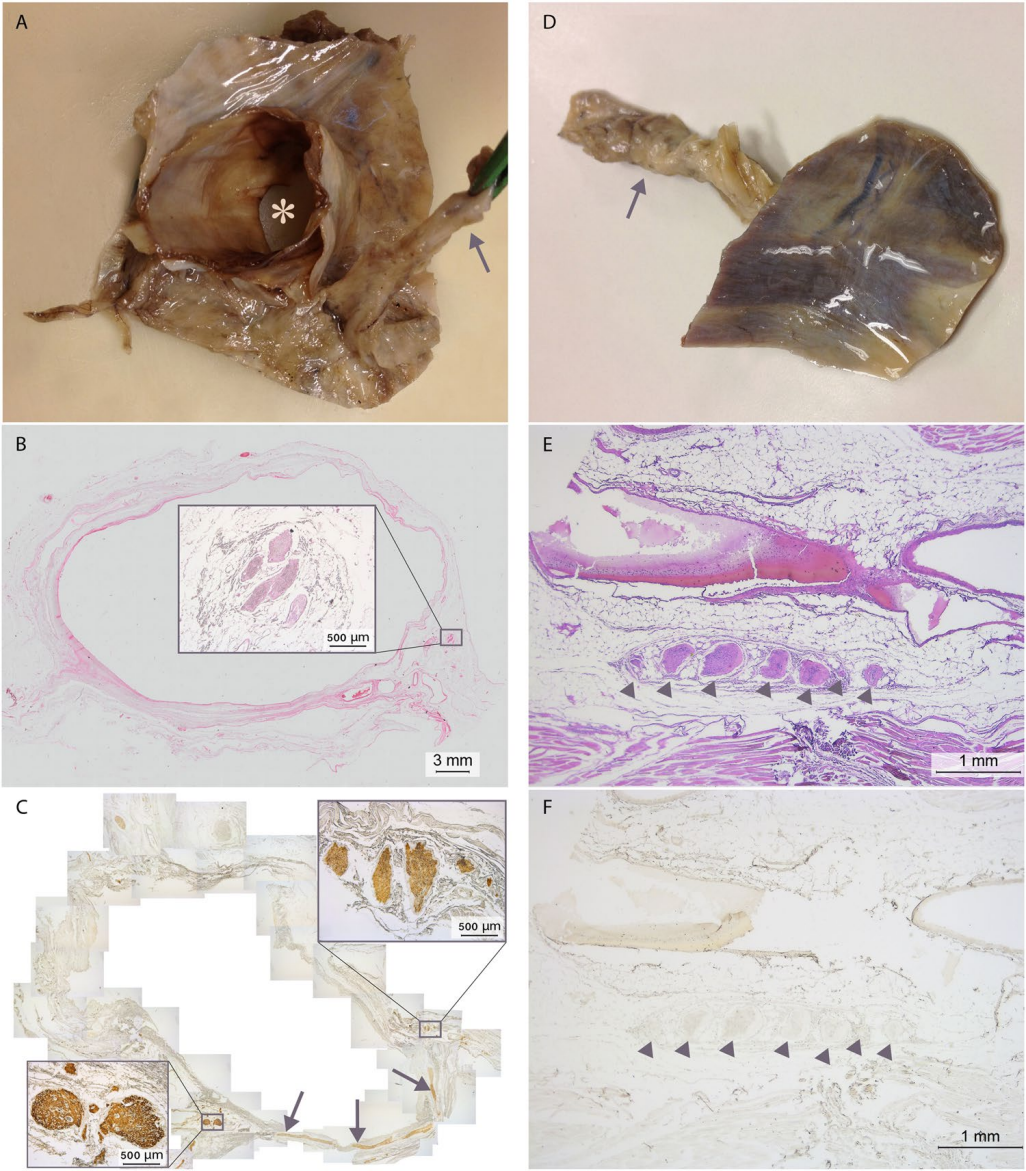
Intradiaphragmatic course of the left and right phrenic nerves. (**A**–**C**) right phrenic nerve passing through the foramen of the inferior caval vein. (**A**) Macroscopic sample, cranial view showing the phrenic nerve (arrow), the inferior caval vein (asterisk) and a part of the surrounding diaphragm. (**B**) HE staining of a section of the caval vein shown in (**A**) with a magnification of the phrenic nerve consisting of several branches shown in the inset. (**C**) S-100 staining to show the nervous connection (arrows) between the phrenic nerve (magnification shown in inset on the right) and caval body (magnification shown in inset on the left (see also Fig. 5)). (**D**–**F**) left phrenic nerve passing through the left dome of the diaphragm. (**D**) macroscopic sample with left phrenic nerve (arrow), caudal view. (**E**) HE-stained section of the sample containing several branches of the phrenic nerve (arrows). (**F**) TH-staining of a serial section of E. Note the absence of TH-positive fibres in this part of the phrenic nerve.

The course of the right phrenic nerve along the foramen of the inferior caval vein was analysed in 4 cadavers on transverse sections of ~5 cm diameter (Fig. 4A,B). The branching pattern of the phrenic nerve was irregular, with TH-positive branches accompanying the motor nerve. The intradiaphragmatic course of the left phrenic nerve was also analysed in 4 cadavers (Fig. 4D–F) and was similar to that of the right phrenic nerve, except that TH-positive fibres were completely absent (Fig. 4E,F).

#### Caval bodies

**Figure 5:**
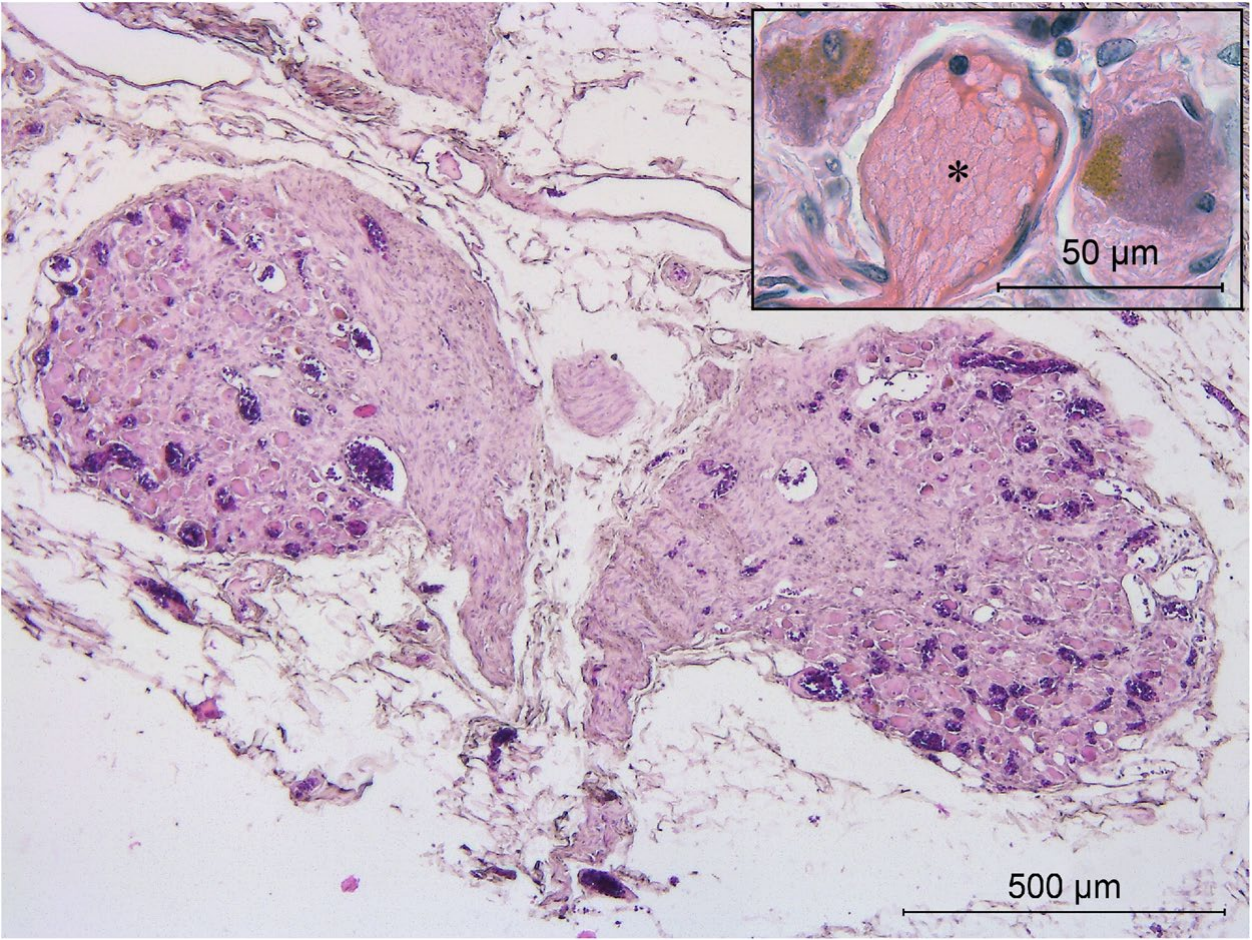
Caval body at the level of the diaphragm. The body consists of nerve fibres (pink), an extensive venous plexus (dark purple), and large cells containing NPPA-positive granules (magnification of a caval body from another specimen is shown in inset, where NPPA-positive cells flank a venule (asterisk)).

**Figure 6:**
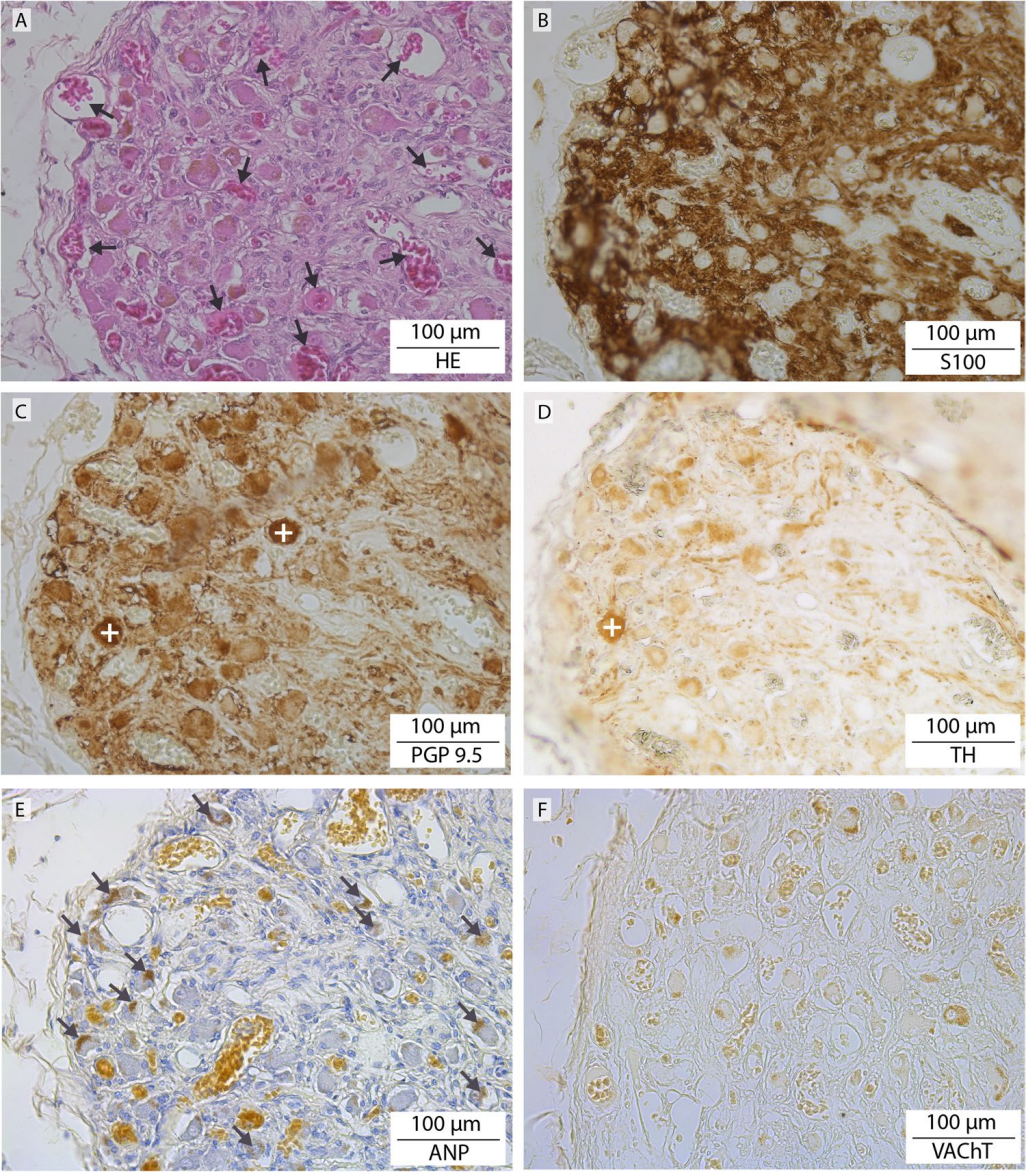
Staining characteristics of a caval body. Serial sections showing a magnified area of the section shown in Fig. 5. Note the extensive venous plexus surrounding cells (**A**, HE; arrows), abundant nerve fibres (**B**, S100), scarce, strongly PGP 9.5-positive (**C**; +) and TH-positive cells (**D**; +) that only partly overlap, and abundant NPPA-positive non-neural cells (**E**; arrows identify intracellular granules; cf. Fig. 5). No VAChT-positive staining was observed (**F**) (visible are some cellular pigments).

In the wall of the inferior caval vein of the 4 cadavers studied, we identified a total of eight tangled structures (Fig. 4C, lower left inset and 5) that contained an extensive venous plexus surrounding large cell bodies with granular inclusions (Fig. 5 inset) and a network of nerve fibres (Figs 5 and 6A,B) originating from the right phrenic nerve on the opposite side (Fig. 4C). Although the large cells morphologically resembled neural cell bodies, only a fraction stained positive for PGP9.5 (Fig. 6C) or TH (Fig. 6D). Neither VAChT- or ChAT-positive cells were observed (Fig. 6F). Many of the large cells bordering the veins (Fig. 5 inset) contained atrial natriuretic peptide (NPPA)-positive granules (Fig. 6E). The phenotypic characteristics of these structures resemble those of paraganglia^21^. For this reason, we have named them “caval bodies”.

#### Myocardial muscle sleeves

In 3 of the 4 cadavers, the wall of the inferior caval vein inside the diaphragm contained longitudinal strands of myocardium that stained positive for α-smooth muscle actin and SERCA2a (supplemental Fig. 1).

### Findings within the abdomen

#### Abdominal course and characteristics of the right phrenic nerve

**Figure 7:**
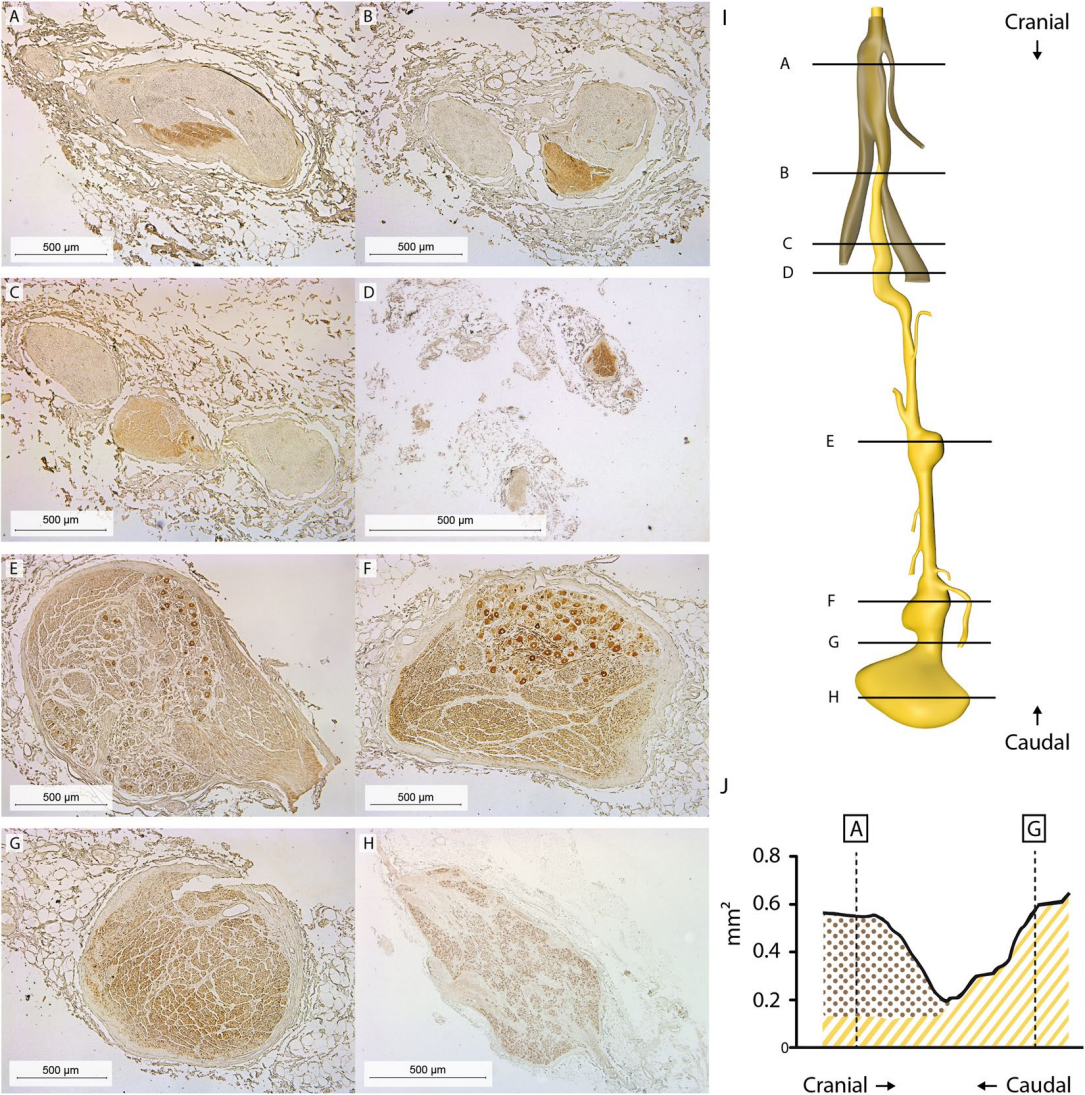
Reconstruction of abdominal portion of the phrenic nerve. 7,500 Sections were sampled every 5 µm. (**A**–**H**) TH-stained sections corresponding to the levels depicted in the reconstruction shown in I (brown: phrenic nerve branches; yellow: phrenic branch of celiac plexus). Phrenic ganglia were found at sites (**E**,**F** and **H**). (**J**) Surface area measurements of the phrenic nerve (brown) and phrenic branch of the celiac plexus (yellow; without ganglia). Illustration drawn by Greet Mommen.

The abdominal course of the right phrenic nerve was studied in 5 cadavers. Textbooks indicate that the nerve extends all the way to the phrenic ganglia in the periphery of the celiac plexus^7^. We tested this assumption by sectioning the abdominal portion of the nerve of one cadaver from the diaphragm to the caudal-most of the phrenic ganglia, (7,500 sections of 5 µm each; Fig. 7A–H). A 3D reconstruction of these sections (Fig. 7I) revealed that the phrenic nerve (Fig. 7A,I (brown)) split into two main, myelinated branches that innervated the diaphragm (Fig. 7B). A TH-positive autonomic fascicle presented as a third, separate branch distal to this level and extended all the way to the celiac ganglion (Fig. 7C–H). We, therefore, named this branch the phrenic branch of the celiac plexus. Of note, the diameter of the branch excluding the ganglia increased towards the celiac ganglion (Fig. 7J) and was interspersed with neuronal cell bodies (Fig. 7E,F,H) that stained positive for PGP9.5, TH and dopamine β-hydroxylase DBH (supplemental Fig. 2).

#### Left and right peri-arterial nerve plexuses of the inferior phrenic artery

Nerve plexuses that accompany the left and right inferior phrenic arteries were studied in 5 cadavers and found to consist of small, purely TH-positive branches in the tunica adventitia. These plexuses, therefore, do not resemble the phrenic branch of the celiac plexus (supplemental Fig. 3).

## Discussion

In this study, we have characterized both phrenic nerves in man with respect to fascicles, fibre composition and myelinisation. We demonstrate that the phrenic nerve also serves as a conduit for catecholaminergic fibres and that the phrenicoabdominal branch of the right phrenic nerve is, instead, a branch of the celiac plexus. The “phrenic branch of the celiac plexus” is, therefore, a more appropriate name for this nerve. In addition, we report the presence of paraganglia (“caval bodies”) and myocardial sleeves in the wall of the inferior caval vein at the level of the diaphragm. The caval bodies were innervated by the right phrenic nerve.

### Morphological characterization of the left and right phrenic nerve

Our data show that the thoracic phrenic nerve generally consists of a single fascicle, which arises from the fusion of several cervical fascicles, and diverges into several fascicles again near the diaphragm. In agreement with an earlier ultrasound study at the cervical level^22^, we found comparable diameters for the left and right human phrenic nerves throughout their course. A small (N = 2) electron-microscopic (EM) study of the phrenic nerve of the rat at the level of the entrance of the inferior caval vein into the right atrium revealed that the right phrenic nerve contained ~30% more axons than its left counterpart^23^.

### TH positive fibres and fascicles

Communicating nerve fibres of the phrenic nerve have been described macroscopically for the somatic subclavian and sternohyoid nerves, the ansa cervicalis, the accessory, supraclavicular, suprascapular and hypoglossal nerves^3^ and the vagus nerve^24^. Communicating fibres with sympathetic nerves include the subclavian ansa, the cervical sympathetic trunk (including the middle and stellate ganglion), and the splanchnic nerves^5,6,8,24–30^. These fibres were also observed in other species^27,29,31,32^ and hypothesized to be vasoregulators of the diaphragmatic vessels^27,33^.

This study histologically validates the presence and composition of TH positive communicating nerve fibres between the right phrenic nerve and celiac plexus (see further). In the supradiaphragmatic part of the right phrenic nerve, the TH positive fibres are present as distinct fascicles or as distinct areas within the phrenic perineurium or epineurium, respectively. Such distinct TH positive areas were only seen in the right phrenic nerve just above the diaphragm, but also in both phrenic nerves in the cervical region. Therefore, we hypothesize that TH-positive fascicles form communications with nearby nerves or organs in this area too.

### Abdominal course and characteristics of the right phrenic nerve

Previous gross-anatomy reports suggest that the phrenic nerve continues on the abdominal side of the diaphragm to the phrenic ganglia^7,9,10^. This branch is classically described as the ‘phrenicoabdominal branch of the right phrenic nerve’^34^. Our 3D reconstruction of the right phrenic nerve and analysis of the composition of the left phrenic nerve revealed that the phrenic nerve motor branches do not continue beyond the diaphragm. Instead, the right phrenic nerve continues as a completely catecholaminergic nerve branch that, based on its increasing diameter, arises from the celiac ganglia and is, therefore, more appropriately termed the ‘phrenic branch of celiac plexus’. TH- and DBH-positive cell bodies were encountered throughout the phrenic branch of celiac plexus, that is, also outside the two macroscopically visible ganglia that are classically described^9,11^. Based on these findings, we conclude that the phrenic motor nerve innervates the diaphragm, but also serves as a conduit for the peripheral autonomic nervous system. The absence of catecholaminergic fibres in the intradiaphragmatic part of the left phrenic nerve emphasizes the asymmetry of the distribution of the autonomic fibres. We hypothesize that this asymmetry corresponds with the presence of paraganglia in the wall of the (right-sided) inferior caval vein (see next paragraph).

### Paraganglia at the level of the diaphragm

An unexpected finding in this study was the identification of paraganglia in the wall of the inferior caval vein where it passed the diaphragm. Based on their morphological appearance, the extensive venous network around NPPA-positive cells and the rich innervation by fibres arising from the phrenic nerve, we hypothesize that these structures have a neuroendocrine function like paraganglia elsewhere. Fibres of the phrenic nerve encircling the inferior caval vein have also been described in the foetus^35^. The presence of NPPA-positive granules further suggests that these cells have a role in regulating plasma volume in a similar manner as NPPA-containing cardiomyocytes. By analogy to the vagal B-type atrial receptors^36^, which monitor central venous pressure as stretch of the atrial wall, the phrenic or autonomic nerve endings could act as low-pressure receptors for the central venous pressure. As elsewhere^37^, the NPPA-positive cells could be under efferent catecholaminergic neural control. It would be interesting to investigate whether right phrenic nerve stimulation affects plasma levels of NPPA (fragments) and influences volume homeostasis. Blood pressure management is a key element in the treatment of patients suffering from HF and many other conditions.

Further characterization of these neuroendocrine structures is desirable, since neuroendocrine, chemosensory and neuroimmunomodulatory functions exist in other paraganglia like the carotid body^21^.

### Myocardial muscle strands

Another unexpected finding was the presence of longitudinal cardiac muscle strands in the wall of the inferior caval vein. A caval sphincter supplied by the right phrenic nerve is a well-known feature of diving mammals^38^. However, this sphincter is usually described as consisting of striated skeletal muscle that is continuous with the diaphragm^38^. The expression of α-smooth muscle actin indicates that the myocardium in these strands is poorly differentiated^39^. Myocardial ‘sleeves’ with such properties have also been described in pulmonary veins and at the base of the pulmonary trunk, where their presence can establish extranodal pacemaker activity^40^.

### Clinical implications

We observed myelinated and non-myelinated nerve fibres in both phrenic nerves without differences between left and right or along the proximo-distal course of the nerves. Such information is important for nerve stimulation, because myelinated nerve fibres have a much lower amplitude-duration threshold upon nerve stimulation than non-myelinated fibres^41,42^. Typical stimulation protocols for (transvenous) phrenic nerve stimulation can vary up to a hundred-fold in intensity (0.1–10 mA), 5-fold in duration (60–300 µs) and 2-fold in frequency (20–40 Hz)^13^. If applied for central sleep apnoea, the stimulation should target the myelinated fibres and should, therefore, be accomplished with the lowest possible amplitude-duration thresholds that result in the intended rhythmic activation of the diaphragm. This is necessary to prevent any undesired stimulation of the nonmyelinated catecholaminergic fibres that are also present within the phrenic nerve. This concern is relevant, because (direct) electric stimulation of the right subclavian ansa did elevate noradrenaline and cAMP concentrations in plasma harvested in the coronary sinus of dogs^43^. Such a catecholaminergic stimulation of the heart may, therefore, further increase the chronic upregulation of sympathetic activity that is already present in patients with central sleep apnoea^44,45^ and that is associated with increased mortality these patients^15–17^.

## Electronic supplementary material

Supplementary Material
