# Supplementary material for: The human phrenic nerve serves as a morphological conduit for autonomic nerves and innervates the caval body of the diaphragm
Source: Sci Rep. 2018 Aug 3;8:11697. doi: 10.1038/s41598-018-30145-x (PMC6076324; doi:10.1038/s41598-018-30145-x)
Supplement: Supplementary file 1 — Supplementary Material [file 41598_2018_30145_MOESM1_ESM.pdf]

The human phrenic nerve serves as a morphological conduit for autonomic nerves and innervates the caval body of the diaphragm

Thomas J.M. Verlinden, Paul van Dijk, Andreas Herrler, Corrie de Gier- de Vries, Wouter H. Lamers, S. Eleonore Köhler.

Supplemental figure 1. Myocardial strands.

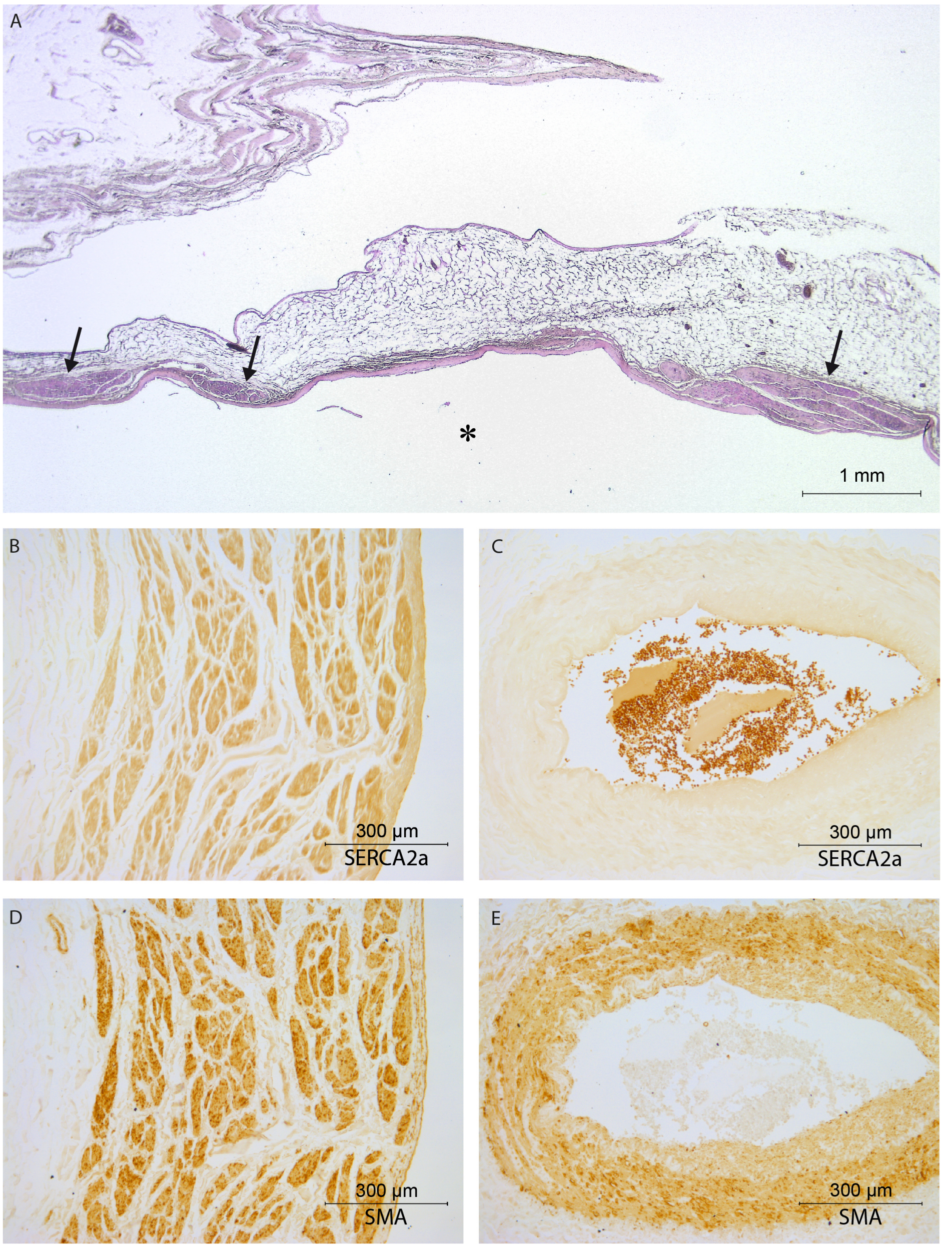

A: HE: Clustered, longitudinally arranged cardiac muscle fibres residing in the wall of the inferior caval vein (arrows) (lumen of inferior caval vein indicated by asterisk). B: SERCA2-positive staining of myocardial strands. C: SERCA2a-negative staining of adjacent arterial smooth muscle wall. D: SMA-positive staining of myocardial strands. E: SMA-positive staining of adjacent arterial smooth muscle wall.

Supplemental figure 2. Ganglion in the phrenic branch of the celiac plexus.

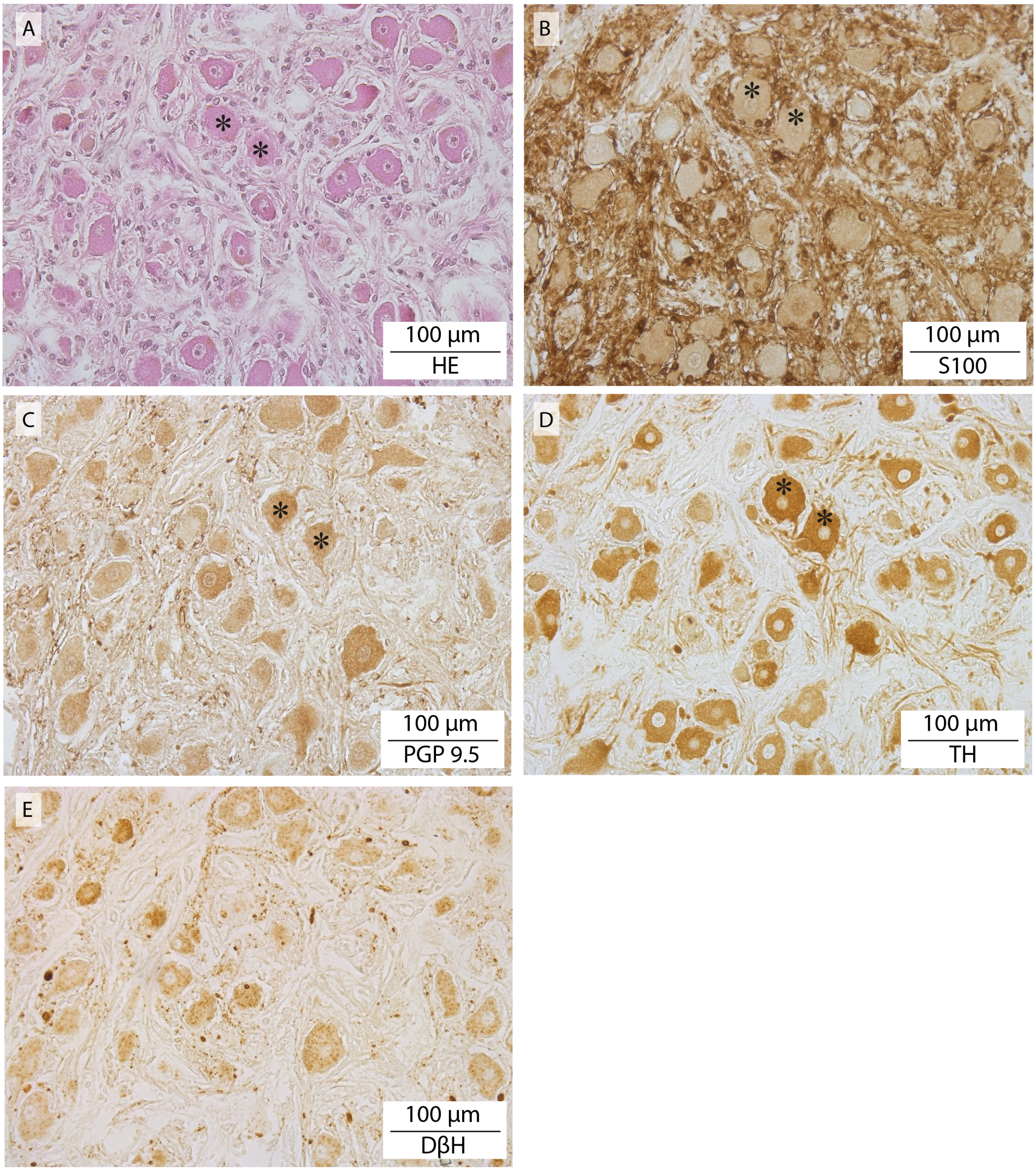

Serial sections stained with A: HE; B: S-100; C: PGP.9.5; D: TH; and E: DBH. Identical cells are indicated with an asterisk.

Supplemental figure 3. Peri-arterial nerve plexus of the inferior phrenic artery.

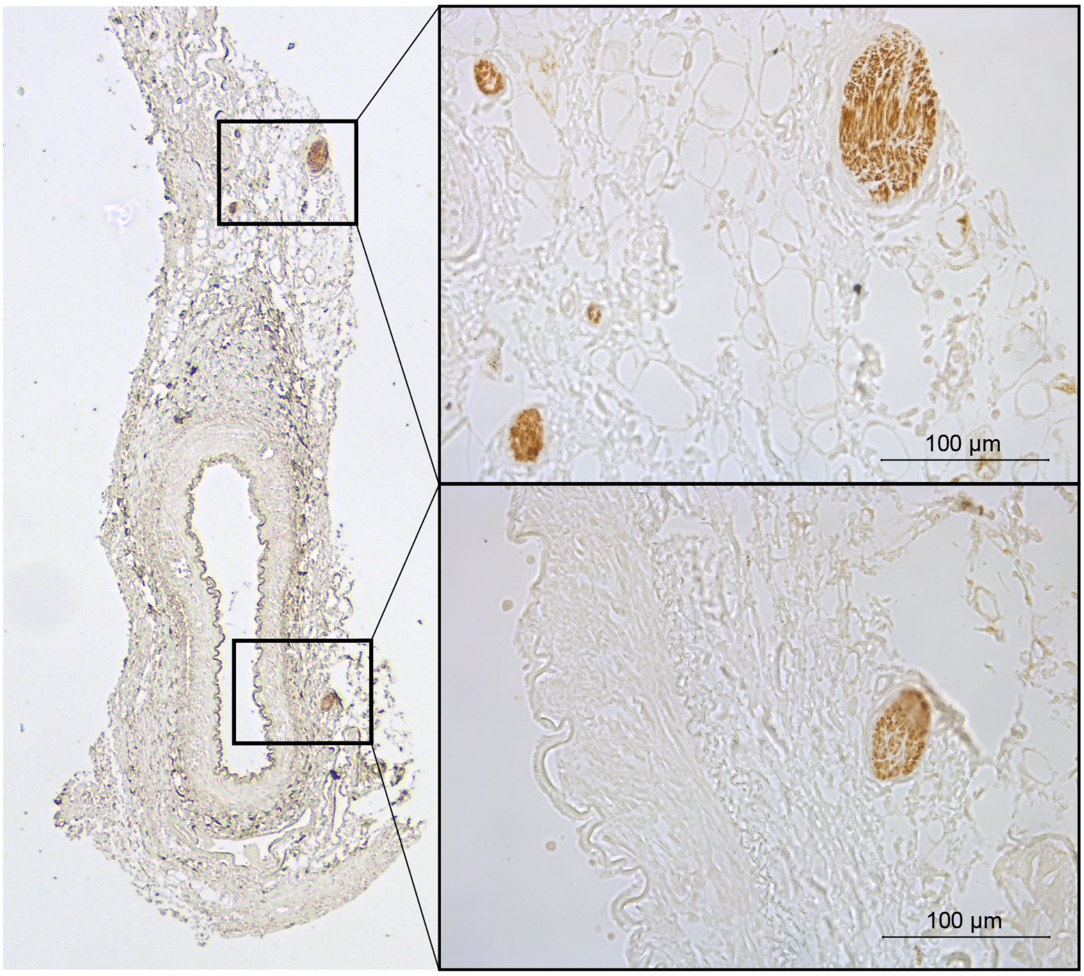

Left inferior phrenic artery stained for the presence of TH
